# Discordance of epidermal growth factor receptor mutation between primary lung tumor and paired distant metastases in non-small cell lung cancer: A systematic review and meta-analysis

**DOI:** 10.1371/journal.pone.0218414

**Published:** 2019-06-19

**Authors:** Chia Ching Lee, Yu Yang Soon, Char Loo Tan, Wee Yao Koh, Cheng Nang Leong, Jeremy Chee Seong Tey, Ivan Weng Keong Tham

**Affiliations:** 1 Department of Radiation Oncology, National University Cancer Institute Singapore, National University Hospital Singapore, Singapore, Singapore; 2 Department of Pathology, National University Hospital Singapore, Singapore, Singapore; Universita degli Studi della Campania Luigi Vanvitelli, ITALY

## Abstract

**Purpose:**

To evaluate the rate of discordance of epidermal growth factor receptor (EGFR) mutation between primary lung tumor and paired distant metastases in non-small-cell lung cancer (NSCLC).

**Methods:**

We performed a meta-analysis of 17 studies (518 cases) assessing discordance rates of EGFR mutation in primary tumors and paired distant metastases. We performed subgroup analyses based on EGFR mutation status in primary tumor (mutant or wildtype), site of distant metastasis (bone, central nervous system (CNS) or lung/ pleural), methods of testing (direct sequencing or allele-specific testing) and timing of metastasis (synchronous or metachronous).

**Results:**

The overall discordance rate in EGFR mutation was low at 10.36% (95% CI = 4.23% to 18.79%) and varied widely between studies (I^2^ = 83.18%). The EGFR discordance rate was statistically significantly higher in bone metastases (45.49%, 95% CI = 14.13 to 79.02) than CNS (17.26%, 95% CI = 7.64 to 29.74; P = 0.002) and lung/ pleural metastases (8.17%, 95% CI = 3.35 to 14.85; P < 0.001). Subgroup analyses did not demonstrate any significant effect modification on the discordance rates by the EGFR mutation status in primary lung tumor, methods of testing and timing of metastasis.

**Conclusion:**

The overall discordance rate in EGFR mutation between primary lung tumor and paired distant metastases in NSCLC is low, although higher discordance rates were observed in bone metastases compared with CNS and lung/pleural metastases. Future studies assessing the impact of EGFR mutation discordance on treatment outcomes are required.

## Introduction

Increased understanding in molecular pathology in advanced non-small cell lung cancer (NSCLC) over the past decades has advocated personalised treatment approaches. Molecular diagnostic testing is now recommended by clinical guidelines for patients with advanced NSCLC to determine eligibility for targeted therapies[1, 2].

Epidermal growth factor receptor (EGFR) mutation is one of the common actionable mutations in advanced NSCLC which is predictive of treatment response to tyrosine kinase inhibitors (TKIs)[3, 4]. Mutation testing can be performed using the samples obtained from the primary lung tumor, metastatic tumor or plasma[5].

Although previous studies have summarized the available literature, a well-conducted systematic review and meta-analysis focusing on the discordance rate of EGFR mutation status between primary tumor and paired distant metastases is lacking[6–8]. The previous reviews included studies evaluating discordance rate between primary tumor and regional lymph nodes and did not assess the methodological quality of the included studies nor summarize the frequency of discordance using meta-analysis techniques.

Hence this study aimed to systematically evaluate the frequency of discordance in EGFR mutation between primary tumor and paired distant metastases among patients with NSCLC in the published peer-reviewed articles and perform a meta-analysis to assess for any differences by metastatic sites, mutation status of primary tumor, methods of testing and timing of metastasis relative to the diagnosis of primary tumor.

## Material and methods

### Study eligibility criteria

We included studies describing molecularly-assessed EGFR mutations involving exons 18, 19, 20 and 21 in primary lung tumors compared with paired distant metastases were included. Exclusion criteria were case reports, meta-analyses, reviews, circulating tumor cells, thoracic lymph nodes or loco-regional metastases and immunohistochemistry (IHC) or fluorescence in situ hybridization FISH) methods for assessing EGFR mutation status.

### Search strategy

The PUBMED and EMBASE databases were searched from their date of inception to 31 May 2018 for relevant studies. The terms of “lung”, “cancer”, “metastasis”, “epidermal growth factor receptor”, “mutation” and “discordance” or “concordance” with their synonyms and MeSH terms, were used for the literature search (S1 Table)

### Study selection and data extraction

**Fig 1:**
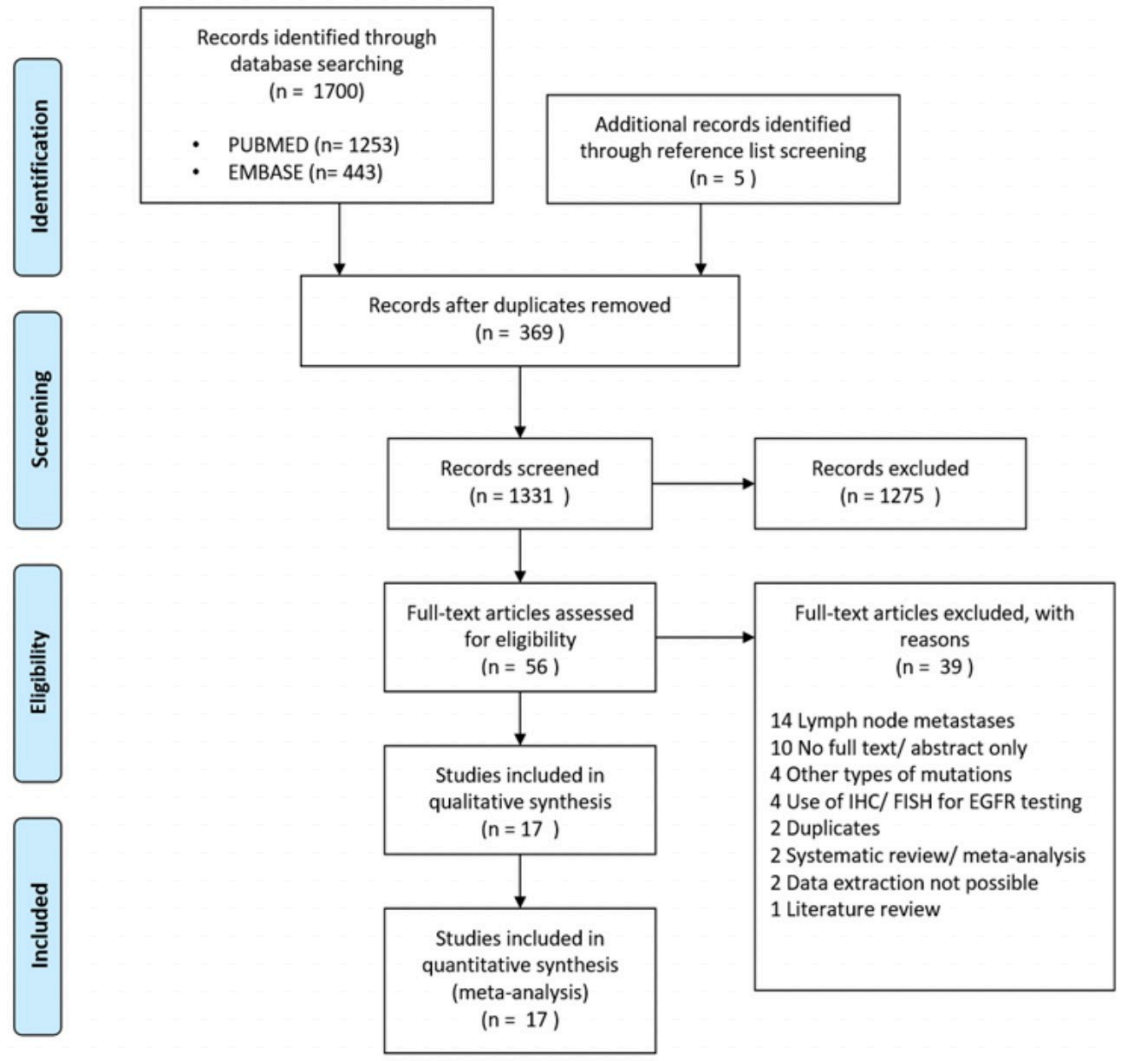
Flow diagram of study selection. Abbreviation: IHC = immunohistochemistry; FISH = fluorescence in-situ hybridization; EGFR = epidermal growth factor receptor.

A total of 1,696 articles were identified. These articles were imported into an online software, COVIDENCE[9] for study screening and selection. Three hundred and sixty-nine duplicates were removed automatically in COVIDENCE. Titles and abstracts were screened for relevance. We excluded 1,275 articles because the title and abstract did not meet the selection criteria. The original full-text papers of 52 articles were extracted and scrutinized. Eventually 13 eligible articles were identified. Reference lists of the papers of interest were screened manually to search for additional relevant studies. Four additional articles were identified. The flow diagram of study selection is illustrated in Fig 1.

Seventeen selected articles were reviewed by two authors to ensure eligibility of each article in the meta-analysis. Quality assessment of these studies was performed using the QUADAS-2 (QUality Assessment of Diagnostic Accuracy Studies) tool[10], with consensus attained between two reviewers (Table 1). This tool consists of four key domains including patient selection, index test, reference standard and flow of patients through the study. Each domain was assessed for risk of bias and the first three were also assessed for concerns regarding applicability.

**Table 1:** Critical appraisal according to QUADAS-2.

Study	Risk of bias				Concerns regarding applicability			
	Patient selection	Index test	Reference standard	Flow and timing	Patient selection	Index test	Reference standard	
Chen et al., 2012[16]	Low risk	Low risk	Low risk	Low risk	Low risk	Low risk	Low risk	Applicable for review
Cortot et al.,2010[17]	Low risk	Low risk	Low risk	Low risk	Low risk	Low risk	Low risk	Applicable for review
Fang et al., 2011[18]	Low risk	Low risk	Low risk	Low risk	Low risk	Low risk	Low risk	Applicable for review
Gow et al., 2009[19]	Low risk	Low risk	Low risk	Low risk	Low risk	Low risk	Low risk	Applicable for review
Han et al., 2011[20]	Low risk	Low risk	Low risk	Low risk	Low risk	Low risk	Low risk	Applicable for review
Kalikaki et al., 2008[21]	Low risk	Low risk	Low risk	Low risk	Low risk	Low risk	Low risk	Applicable for review
Kamila et al., 2013[22]	Low risk	Low risk	Low risk	Low risk	Low risk	Low risk	Low risk	Applicable for review
Liu et al., 2018[23]	Low risk	Low risk	Low risk	Low risk	Low risk	Low risk	Low risk	Applicable for review
Luo et al., 2014[24]	Low risk	Low risk	Low risk	Low risk	Low risk	Low risk	Low risk	Applicable for review
Mansuet-Lupos et al., 2014[25]	Low risk	Low risk	Low risk	Low risk	Low risk	Low risk	Low risk	Applicable for review
Matsumoto et al., 2006[26]	Low risk	Low risk	Low risk	Low risk	Low risk	Low risk	Low risk	Applicable for review
Monaco et al., 2010[27]	Low risk	Low risk	Low risk	Low risk	Low risk	Low risk	Low risk	Applicable for review
Quere et al., 2016[28]	Low risk	Low risk	Low risk	Low risk	Low risk	Low risk	Low risk	Applicable for review
Rau et al., 2016[29]	Low risk	Low risk	Low risk	Low risk	Low risk	Low risk	Low risk	Applicable for review
Sun et al., 2009[30]	Low risk	Low risk	Low risk	Low risk	Low risk	Low risk	Low risk	Applicable for review
Takahashi et al., 2007[31]	Low risk	Low risk	Low risk	Low risk	Low risk	Low risk	Low risk	Applicable for review
Yatabe et al., 2011[32]	Low risk	Low risk	Low risk	Low risk	Low risk	Low risk	Low risk	Applicable for review

Abbreviations: QUADAS = Quality assessment of diagnostic accuracy studies

The data extracted from the studies, includes year of article publication, total number of patients evaluated, EGFR mutation status of primary and distant metastases, site of metastasis, method of testing, timing of metastasis relative to the diagnosis of primary tumor, specimen for testing (histology or cytology), study design as well as the region where the study was conducted.

The Preferred Reporting Items for Systematic Reviews and Meta-Analyses (PRISMA) statement checklist was used for transparent reporting of the study selection process[11].

### Outcome of interest

The outcome of interest in this study was the discordance rate of EGFR mutation status between primary lung tumor and paired distant metastases. We defined discordance as a change of mutation status from mutant to wild-type or vice versa. A change of a positive EGFR mutation to a different type of positive EGFR mutation was not considered discordant, for example, a case of EGFR exon 19 deletion in primary lung tumor with exon 21 point mutation in paired distant metastasis was not taken as a discordance.

### Statistical analysis

The percentages of EGFR mutation discordance and 95% confidence intervals (CIs) were calculated for each study. Subgroup analyses were performed for EGFR mutation status of primary tumor (mutant vs wild-type), site of metastasis (bone, central nervous system (CNS), lung/ pleural or others), testing methodology (direct sequencing or allele-specific testing) and timing of metastasis (synchronous or metachronous).

For meta-analysis, we use the Freeman-Tukey arcsine square root transformation to calculate the weighted summary proportion under the random effects model[12, 13]. Heterogeneity across studies was assessed using Q test and I^2^ methods[14, 15]. I^2^ values of 0%, 25%, 50% and 75% are indicated as “no”, “low”, “moderate” and “high” heterogeneity. Comparison of subgroups was performed using chi-square test.

The statistical analyses were performed using MedCalc statistical software version 18.0. P-values of less than 0.05 were considered statistically significant.

### Sensitivity analysis

We have conducted a post-hoc sensitivity analysis as suggested by the reviewers to exclude two studies (Kalikaki et al., 2008 and Gow et al., 2009)[16, 17] as the assays used in these studies may be unreliable.

## Results and discussion

### Study characteristics

We identified 17 eligible studies with a total of 518 patients[16–32]. The characteristics of the studies are summarized in Table 2. The median sample size was 21. The most common reported site of distant metastasis was lung/ pleura (51%, 264/518), followed by CNS (22%, 116/518) and bone (4%, 21/518). Almost half of population had EGFR-mutant primary tumors (51%, 163/518). About half of the patients were tested using allele-specific testing (55%, 284/518). Most of the studies (13 out of 17) did not report or categorize whether the metastases were synchronous or metachronous. All the seventeen studies were judged to have low risk of methodological bias and applicable for review (Table 1). Comparison between primary lung tumors and paired distant metastases was summarized in S2 Table.

**Table 2:** Characteristics of eligible studies.

Study	Country	Study design	Number of eligible subjects (N = 518)	Age at diagnosis in year (range; median)	Site of metastasis n, (%)				EGFR Mutation status of primary tumor n, (%)						Methods of Testing n, (%)		Timing of Metastasis n, (%)	
									EGFR Mutant					EGFR Wild-type	Direct sequencing	Allele-specific testing	Synchronous	Metachronous
					Bone	CNS	Lung/ Pleura	Others^¥^	Exon 18	Exon 19	Exon 20	Exon 21	Total					
Chen et al., 2012[16]	China	R	35	NR	NR				0 (0)	NR			16 (46)	19 (54)	35 (100)	0 (0)	NR	
Cortot et al.,2010[17]	France	R	19	NR	NR				0 (0)	0 (0)	0 (0)	0 (0)	0 (0)	19 (100)	NR	0 (0)	NR	
Fang et al., 2011[18]	China	R	4	NR	0 (0)	4 (100)	0 (0)	0 (0)	0 (0)	0 (0)	0 (0)	1 (100)	1 (25)	3 (75)	0 (0)	4 (100)	NR	
Gow et al., 2009[19]	Taiwan	R	48*	NR	17 (35)	20 (42)	1 (2)	10 (21)	0 (0)	16^^^ (59)	0 (0)	10 (37)	27 (56)	21 (44)	28 (58)	20 (42)	NR	
Han et al., 2011[20]	South Korea	R	32	NR	0 (0)	5 (16)	23 (72)	4 (13)	1 (6)	6 (38)	0 (0)	9 (56)	16 (50)	16 (50)	32 (100)	0 (0)	28 (87.5)	4 (12.5)
Kalikaki et al., 2008[21]	Greece	R	20	41–70; 54.5	2 (10)	3 (15)	9 (45)	5 (25)	1 (20)	3 (60)	0 (0)	1 (20)	5 (25)	15 (75)	20 (100)	0 (0)	0 (0)	20 (100)
Kamila et al., 2013[22]	Poland	R	2	46–55; 50.5	0 (0)	2 (100)	0 (0)	0 (0)	0 (0)	1 (50)	0 (0)	1 (50)	2 (100)	0 (0)	0 (0)	2 (100)	2 (100)	0 (0)
Liu et al., 2018[23]	China	R	189	NR	0 (0)	0 (0)	189 (100)	0 (0)	NR				116 (61)	73 (39)	0 (0)	189 (100)	NR	
Luo et al., 2014[24]	China	R	15	38–74; 55.0	0 (0)	15 (100)	0 (0)	0 (0)	0 (0)	4 (57)	0 (0)	3 (43)	7 (47)	8 (53)	0 (0)	15 (100)	NR	
Mansuet-Lupos et al., 2014[25]	France	R	2	NR	0 (0)	0 (0)	2 (100)	0 (0)	0 (0)	0 (0)	0 (0)	0 (0)	0 (0)	2 (100)	0 (0)	2 (100)	NR	
Matsumoto et al., 2006[26]	Japan	R	6	43–70; 51.5	0 (0)	6 (100)	0 (0)	0 (0)	0 (0)	5 (83)	0 (0)	1 (17)	6 (100)	0 (0)	6 (100)	0 (0)	0 (0)	6 (100)
Monaco et al., 2010[27]	USA	R	40	42–85; 65.0	NR				0 (0)	0 (0)	0 (0)	0 (0)	0 (0)	40 (100)	40 (100)	0 (0)	NR	
Quere et al., 2016[28]	France	R	21	NR	2 (9)	7 (33)	9 (43)	3 (14)	0 (0)	3 (75)	0 (0)	1^#^ (25)	4 (19)	17 (81)	0 (0)	0 (0)	NR	
Rau et al., 2016[29]	China	R	49	46–86; 63.0	0 (0)	49 (100)	0 (0)	0 (0)	0 (0)	13^@^ (43)	0 (0)	17 (57)	30 (61)	19 (39)	0 (0)	49 (100)	NR	
Sun et al., 2009[30]	USA	R	1	NR	0 (0)	1 (100)	0 (0)	0 (0)	0 (0)	1 (100)	0 (0)	0 (0)	1 (100)	0 (0)	1 (100)	0 (0)	NR	
Takahashi et al., 2007[31]	Japan	R	3	NR	0 (0)	3 (100)	0 (0)	0 (0)	0 (0)	0 (0)	0 (0)	0 (0)	0 (0)	3 (100)	0 (0)	3 (100)	NR	
Yatabe et al., 2011[32]	Japan	R	32	NR	0 (0)	1 (3)	31 (97)	0 (0)	NR				32 (100)	0 (0)	32 (100)	0 (0)	NR	

Abbreviations: EGFR = epidermal growth factor receptor, CNS = central nervous system, R = retrospective, NR = not reported

Others^**¥**^ = liver, adrenal, skin, intestine, pericardium, ovary

*20 cases were duplicated as the primary lung tumor and paired metastases were tested using both direct sequencing and allele-specific testing.

^^^One case had two mutations (exon 19 deletion and L858R). One case had three mutations (exon 19 deletion, L858R and T790M).

^#^One case had two mutations (L858R and T790M).

^@^Three cases had two mutations (exon 19 deletion and L858R).

### Discordance in EGFR mutation between primary and distant metastases

**Fig 2:**
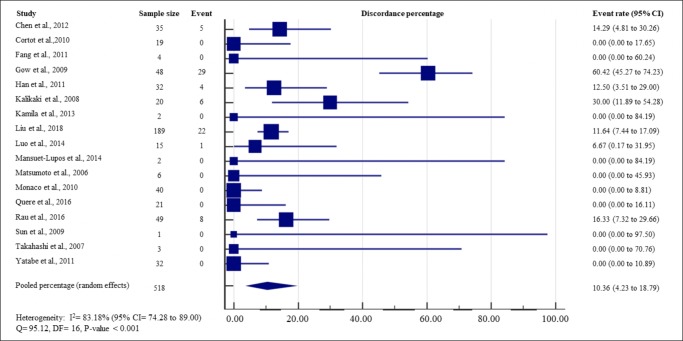
Study-specific and pooled estimate for EGFR mutation discordance percentages. Heterogeneity was assessed using I^2^ and Q test. Event was defined as discordance of EGFR mutation status between primary lung tumor and paired metastases. Abbreviation: DF = degree of freedom; CI = Confidence interval; EGFR = epidermal growth factor receptor.

The total percentage of discordance in EGFR mutation between primary lung tumor and paired distant metastases varied between studies from 0.00 to 60.42%, with a pooled random effects percentage of 10.36% (95% CI = 4.23 to 18.79) (Fig 2). The heterogeneity between studies was high for overall EGFR discordance rate (Q test P-value < 0.001, I^2^ = 83.18%, 95% CI = 74.28 to 89.00).

### Discordance in EGFR mutation between primary and distant metastases by site of metastases

**Fig 3:**
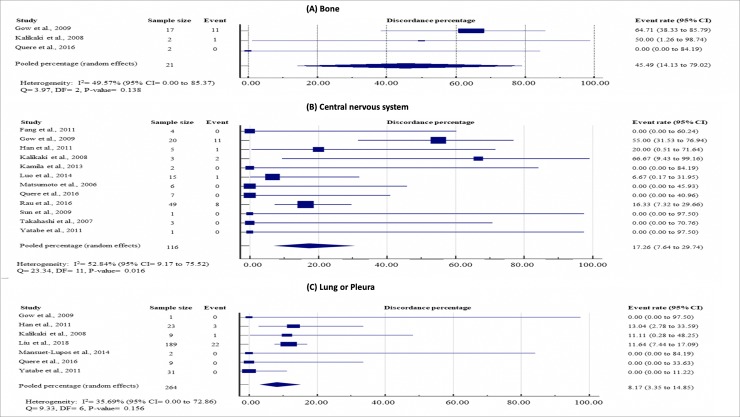
Study-specific and pooled estimates for metastasis location-specific EGFR mutation discordance percentages. **(A) Bone; (B) central nervous nystem; and (C) lung or pleura.** Heterogeneity was assessed using I^2^ and Q test. Event was defined as discordance of EGFR mutation status between primary lung tumor and paired metastases. Abbreviation: DF = degree of freedom; CI = Confidence interval; EGFR = epidermal growth factor status.

Analyses were performed with subgroups representing the most frequent sites of distant metastases. CNS, lung/ pleural metastases and bone were described in twelve, seven and three studies, respectively. The discordance rates in EGFR mutation between primary and metastatic tumors for each metastatic site were summarized in Fig 3. A statistically significant difference in EGFR discordance was observed between all metastatic subsites (P < 0.001), with bone metastases (45.49%, 95% CI = 14.13 to 79.02) having significantly higher discordance rates than CNS (17.26%, 95% CI = 7.64 to 29.74; P = 0.002) and lung/ pleural metastases (8.17%, 95% CI = 3.35 to 14.85; P < 0.001). There was also significantly higher discordance rates between primary lung tumor and CNS metastases compared with lung/ pleural metastases (P = 0.007). Among the eleven discordant cases in bone metastases, five of them had EGFR mutation in primary lung tumors. (S3 Table).

Due to small sample sizes and lack of relevant details in some articles, metastatic sites such as liver, adrenal glands and skin, were not analysed.

### Discordance in EGFR mutation between primary and distant metastases by mutation status of primary tumors, testing methodology and timing of metastasis

**Fig 4:**
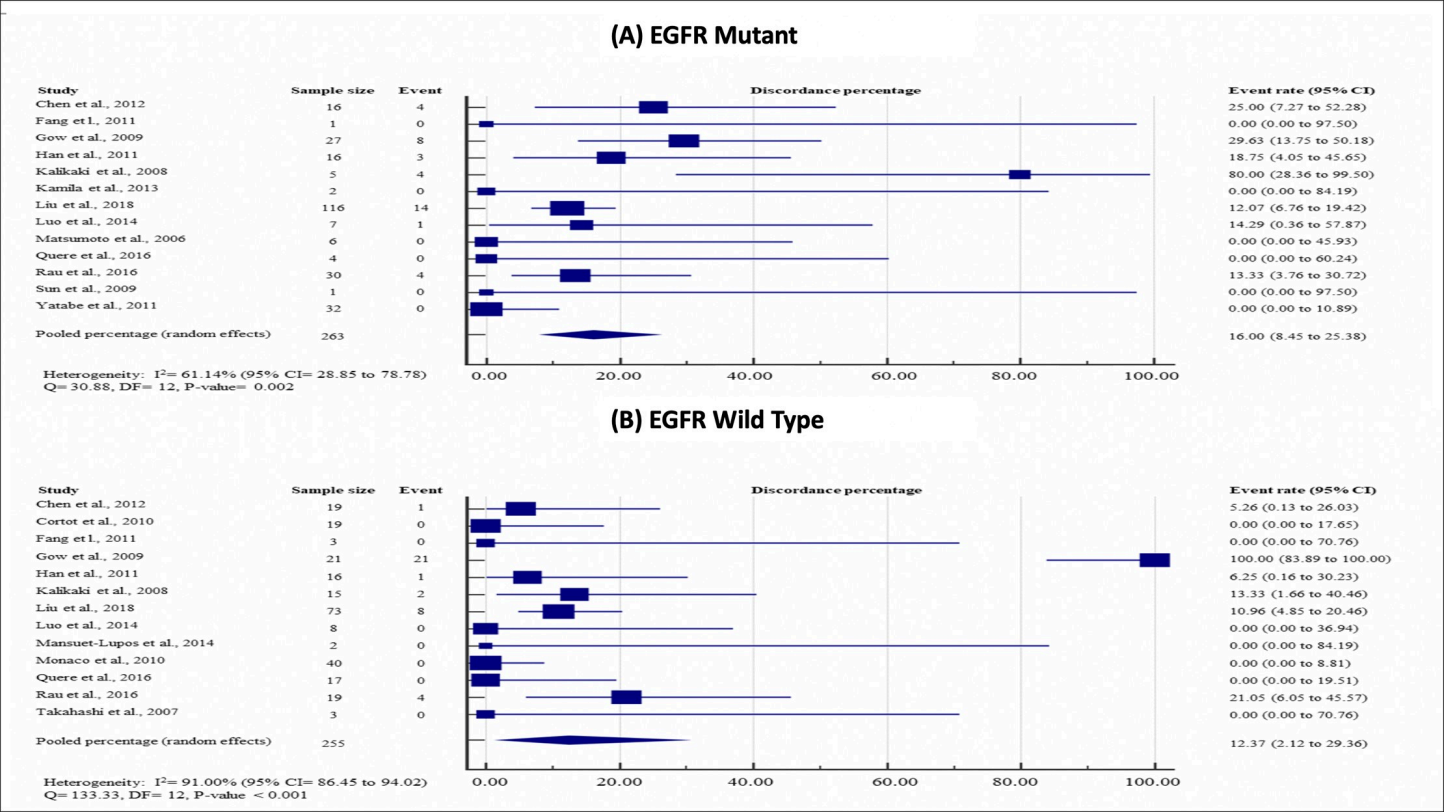
Study-specific and pooled estimates for EGFR mutation discordance percentages by mutation status of primary tumor. **(A) EGFR mutant; and (B) EGFR wild-type.** Heterogeneity was assessed using I^2^ and Q test. Event was defined as discordance of EGFR mutation status between primary lung tumor and paired metastases. Abbreviation: DF = degree of freedom, CI = Confidence interval, EGFR = epidermal growth factor status.

**Fig 5:**
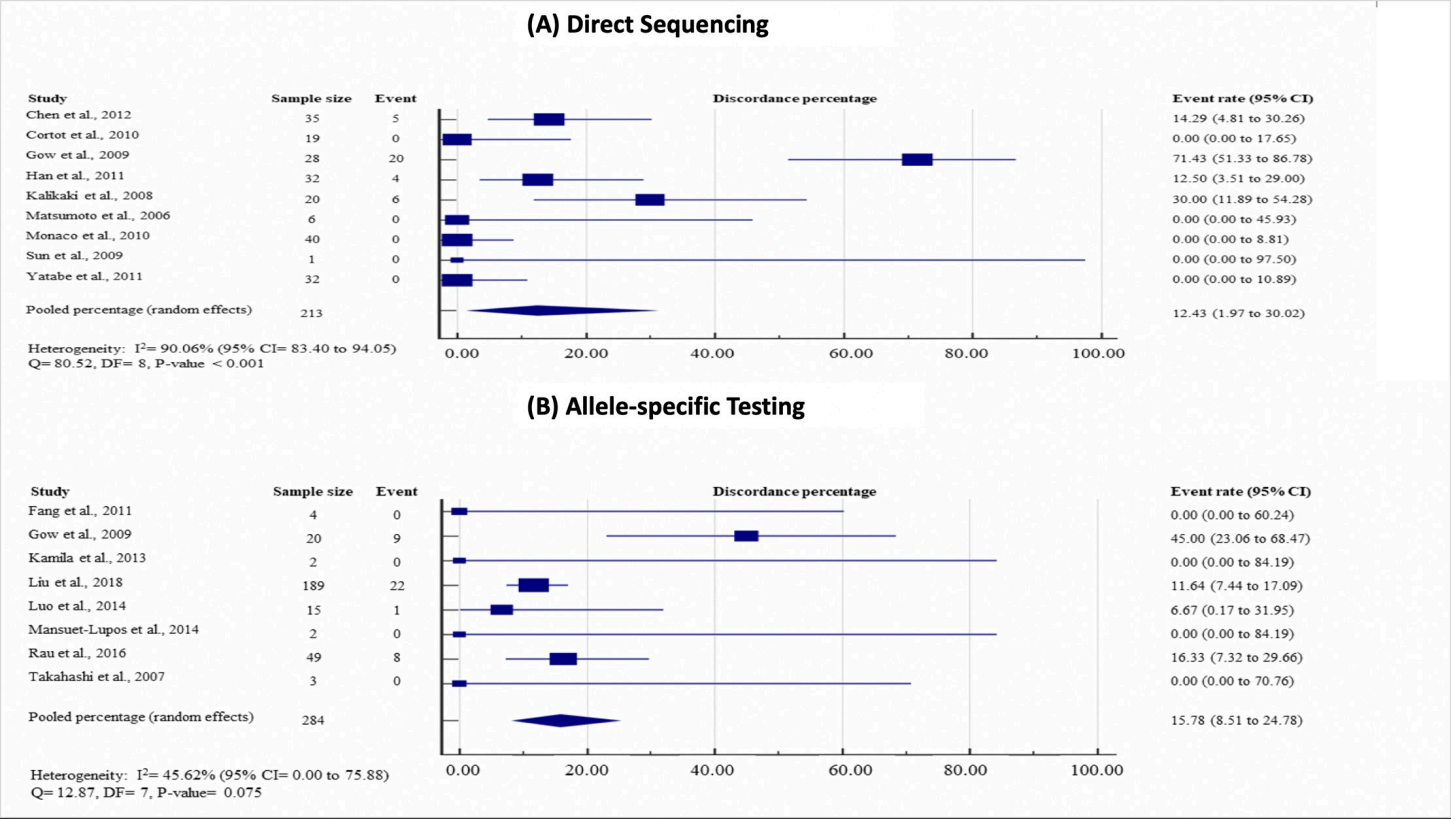
Study-specific and pooled estimates for EGFR mutation discordance percentages by testing methods. **(A) Direct sequencing; and (B) allele-specific testing.** Heterogeneity was assessed using I^2^ and Q test. Event was defined as discordance of EGFR mutation status between primary lung tumor and paired metastases. Abbreviation: DF = degree of freedom, CI = Confidence interval, EGFR = epidermal growth factor status.

**Fig 6:**
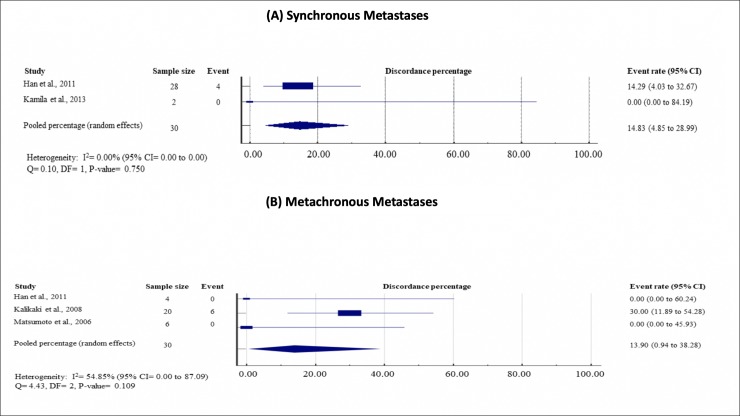
Study-specific and pooled estimates for EGFR mutation discordance percentages by timing of metastases. **(A) Synchronous metastases; and (B) metachronous metastases.** Heterogeneity was assessed using I^2^ and Q test. Event was defined as discordance of EGFR mutation status between primary lung tumor and paired metastases. Abbreviation: DF = degree of freedom, CI = Confidence interval, EGFR = epidermal growth factor status.

Subgroup analyses did not show any significant effect modifications on the EGFR discordance rates by EGFR mutation status of primary tumor (mutant, 16.00%, 95% CI = 8.45 to 25.38; wild-type, 12.37%, 95% CI = 3.53 to 29.64; P = 0.267), testing methods (direct sequencing, 12.43%, 95% CI = 1.97 to 30.02; allele-specific testing, 15.78%, 95% CI = 8.51 to 24.78; P = 0.252) and timing of metastasis (synchronous, 14.83%, 95% CI = 4.85 to 28.99; metachronous, 13.90%, 95% CI = 0.94 to 38.28; P = 0.720). (see Figs 4–6, which demonstrate study-specific and pooled estimates for EGFR mutation discordance percentages by mutation status of primary tumor, testing methods and timing of metastases, respectively).

### Sensitivity analysis

Exclusion of the two studies (Kalikaki et al., 2008 and Gow et al., 2009)[16, 17] shifted the overall discordance rate from 10.36% (95% CI = 4.23 to 18.79) to 6.73% (95% CI = 3.34 to 11.29).

## Discussion

This study demonstrated that the overall discordance rates in EGFR mutation between primary and distant metastases in NSCLC was low but varied largely among the included studies. In addition, metastasis site-specific differences were found, with significantly higher EGFR mutation discordance in bone compared to CNS and lung/ pleural metastases.

The findings of this study are comparable with previous reviews[6–8]. Han et al identified seven retrospective studies which tested for EGFR mutations in exon 19 and 21 in primary lung tumor, regional lymph nodes and distant metastases and reported an overall discordance rate of 17.09%[6]. Wang el al. reviewed nine studies comparing EGFR mutation status between primary and matched lymph node metastases in NSCLC and found that the overall discordance rate of 12.2%[7]. Both studies obtained the discordance rate by summing the total number of discordance cases divided the total number of cases reviewed without using any meta-analysis techniques and included matched lymph node metastases. Sherwood et al. qualitatively reviewed 17 studies reporting on the discordance on EGFR mutation status between primary lung tumor, lymph nodes and distant metastases and found that the discordance rate ranged from 0 to 28%[8].

There are several plausible explanations for the wide variation in discordance rates among the included studies. As shown in the subgroup analyses, the site of distant metastases is the most likely reason contributing to the heterogeneity in the discordance rates among the included studies with studies of bone metastases showing the highest discordance rates. Bone is a complex microenvironment which is relatively resistant to foreign cell settlement and hence could acquire activation of numerous molecular mechanisms for progenitor neoplastic cells to disseminate from bloodstream to skeletal tissue[33, 34]. It is possible that there is a conversion of EGFR mutation status during this process, thus explaining the high discordance rates. Another reason is the diagnostic challenges associated with bone fragmentation, crush artefact and trabecular distortion during sampling procedure. In addition, processing of the bone specimens often requires decalcification reagents which usually contain strong acid that can damage the nucleic acid, resulting in degraded DNA material and a consequent higher discordance rates[35, 36].

Several models of tumor progression were proposed, including clonal evolution/ selection, parallel development and the same gene models[37, 38]. Discordance between primary and metastatic tumors can be explained by both parallel development and clonal selection models. The former model predicts early generation of disseminated cancel cells to distant organs with highly diverse genetic profiles of the primary and metastasis and hence serves as an explanation for the discordance seen in synchronous tumor. Clonal selection during the multistep metastatic progression, with a potential influence of microenvironment and/ or treatment effects[39, 40] will explain for the discordance seen in metachronous tumors. Metastatic relapsing tumor may have acquired new genetic mutations or developed resistance (for example, T970M) along the metastatic process[41, 42]. Our study did not demonstrate that the timing of metastases modified the EGFR discordance rates between primary lung and distant metastases. On the other hand, the concordance cases are likely to be explained by the same-gene model, which suggests that metastases occur as late event of tumor progression, and therefore genetic diversity of the metastases is suggested to be minimal. Our findings support all three progression models in NSCLC and suggest the need of vigorous attempt to identify EGFR mutation by testing all primary and metastatic tumors, regardless of the timing of metastases and the EGFR mutation status in the primary tumors, so that not to jeopardize the patients from eligible treatments.

Our study has several limitations and attempts were made to mitigate their confounding effects. Firstly, the results were heterogeneous among the included studies. We attempt to mitigate this by pre-specifying our subgroup analyses and select a group of studies as homogenous as possible by excluding studies which assessed the discordance between primary tumors and corresponding lymph node metastases[43–45] and those which utilized non-standard methods for EGFR testing, like IHC or FISH[46]. Secondly, publication bias may be present. We have attempted to mitigate this by screening two large medical databases, namely PUBMED and EMBASE as well as the references of relevant articles. Thirdly, we did not have the individual patient data of the included studies, thus we are unable to perform more granular analyses to determine how the exposure to systemic therapies could affect the discordance rates between primary lung tumor and metachronous distant metastases.

This study has a few strengths. Firstly, the results of this study are consistent with previous reviews[6–8]. Secondly, this study included four recent studies[23, 28, 29, 47] not covered by previous reviews[6–8]. Lastly, this study employed meta-analysis techniques to summarize the frequency of discordance and investigate which subgroups had the highest discordance rates.

The results of this study have several implications on clinical practice. Firstly, in patients with Stage IV NSCLC without bone metastases, testing the primary lung tumor alone for EGFR mutation is likely enough. Secondly, in patients with bone metastases, testing both the primary tumor and the bone metastases for EGFR mutation status could be considered due to the possible high discordance rate. Thirdly, EGFR mutation is a predictor of treatment response towards EGFR TKI. The discordance in EGFR status may represent the heterogeneity of tumor clones, which could reflect different treatment responses in different individual patients. We believe this will impact on clinical decision and guide clinicians in patient selection, for instance, combination of TKI and chemotherapy for patients should be considered for patients with discordance in EGFR mutant status and paired distant metastases. The survival benefits of this combined approach demonstrated in the recent two phase-3 randomised trials comparing gefitinib plus platinum-based doublet chemotherapy verses gefitinib monotherapy are possibly explained by this biological basis [48, 49].

## Conclusions

In conclusion, the overall discordance rates in EGFR mutation status between primary NSCLC and distant metastatic tumors is low but varied largely between studies. Discordance occurred more commonly in bone compared with brain and lung/ pleural metastatic sites. Future researches assessing the impact of EGFR mutation discordance on treatment efficacy and survival are required.

## Supporting information

S1 Table Search strategy. (PDF)

S2 Table Comparison between primary lung tumors and paired distant metastases. (PDF)

S3 Table EGFR mutation status of primary lung tumors by sites of metastases for discordant cases. (PDF)
