# Supplementary material for: Discordance of epidermal growth factor receptor mutation between primary lung tumor and paired distant metastases in non-small cell lung cancer: A systematic review and meta-analysis
Source: PLoS One. 2019 Jun 19;14(6):e0218414. doi: 10.1371/journal.pone.0218414 (PMC6583965; doi:10.1371/journal.pone.0218414)
Supplement: S1 Table — (PDF) [file pone.0218414.s001.pdf]

S1 Table. Search strategy

Database	Search term
MEDLINE<br>(via PUBMED)	("Lung Neoplasms"[Mesh] OR (thoracic[tiab] OR lung[tiab] OR bronchi*[tiab] OR pulmonary[tiab]) AND (cancer*[tiab] OR carcinoma*[tiab] OR tumor*[tiab] OR tumour*[tiab] OR metatstas*[tiab] OR metastatic[tiab])) AND ("Receptor, Epidermal Growth Factor"[Mesh] OR epidermal growth factor receptor*[tiab] OR EGFR[tiab]) AND ("Mutation"[Mesh] OR "DNA Mutational Analysis"[Mesh] OR mutation[tiab] OR mutations[tiab] OR mutated[tiab] OR mutational[tiab]) AND (discordant[tiab] OR discordance[tiab] OR concordance[tiab] OR concordant[tiab] OR conversion*[tiab] OR convert[tiab] OR converts[tiab] OR converted[tiab] OR matched[tiab] OR match[tiab] OR matching[tiab])
EMBASE	('lung cancer'/exp OR (thoracic:ab,ti OR lung:ab,ti OR bronchi*:ab,ti OR pulmonary:ab,ti) AND (cancer*:ab,ti OR carcinoma*:ab,ti OR tumor*:ab,ti OR tumour*:ab,ti OR metatstas*:ab,ti OR metastatic:ab,ti)) AND ('epidermal growth factor receptor'/exp OR "epidermal growth factor receptor*":ab,ti OR EGFR:ab,ti) AND ('gene mutation'/exp OR 'mutational analysis'/exp OR mutation:ab,ti OR mutations:ab,ti OR mutated:ab,ti OR mutational:ab,ti) AND (discordant:ab,ti OR discordance:ab,ti OR concordance:ab,ti OR concordant:ab,ti OR conversion*:ab,ti OR convert:ab,ti OR converts:ab,ti OR converted:ab,ti OR matched:ab,ti OR match:ab,ti OR matching:ab,ti)
