# Supplementary material for: Discordance of epidermal growth factor receptor mutation between primary lung tumor and paired distant metastases in non-small cell lung cancer: A systematic review and meta-analysis
Source: PLoS One. 2019 Jun 19;14(6):e0218414. doi: 10.1371/journal.pone.0218414 (PMC6583965; doi:10.1371/journal.pone.0218414)
Supplement: S2 Table — (PDF) [file pone.0218414.s002.pdf]

S2 Table. Comparison between primary lung tumors and paired distant metastases

Study	Time between primary lung tumors and paired distant metastases in months (range; median)	Testing of EGFR mutation in primary lung tumors and paired distant metastasis using same test at the same time	Masking of interpretation of primary lung tumrs and paired distant metastases
Chen et al., 2012	NR	Yes	NR
Cortot et al.,2010	NR	Yes	NR
Fang et al., 2011	NR	Yes	NR; 2 observers
Gow et al., 2009	NR	Yes	Yes; 2 observers
Han et al., 2011	NR	Yes	NR
Kalikaki et al., 2008	2-143; 22	Yes	NR; 1 observer
Kamila et al., 2013	NR	Yes	NR; 2 observers
Liu et al., 2018	NR	Yes	NR
Luo et al., 2014	NR	Yes	NR
Mansuet-Lupos et al., 2014	NR	Yes	NR; 2 observers
Matsumoto et al., 2006	0.5-40; 16	Yes	NR
Monaco et al., 2010	12-28; 19	Yes	NR
Quere et al., 2016	NR	Yes	NR
Rau et al., 2016	NR	Yes	NR
Sun et al., 2009	NR	Yes	NR
Takahashi et al., 2007	NR	Yes	NR
Yatabe et al., 2011	0-99; NR	Yes	NR

Abbreviation: EGFR= epidermal growth factor receptor, NR= not reported
