# Supplementary material for: Discordance of epidermal growth factor receptor mutation between primary lung tumor and paired distant metastases in non-small cell lung cancer: A systematic review and meta-analysis
Source: PLoS One. 2019 Jun 19;14(6):e0218414. doi: 10.1371/journal.pone.0218414 (PMC6583965; doi:10.1371/journal.pone.0218414)
Supplement: S3 Table — (PDF) [file pone.0218414.s003.pdf]

S3 Table. EGFR mutation status of primary lung tumors by sites of metastases for discordant cases

Study	Sites of metastases	Total number of discordance	EGFR mutation status of primary lung tumors	
			EGFR mutant	EGFR wild-type
Gow et al., 2009	Bone	11	5	6
	CNS	11	2	9
Han et al., 2011	CNS	1	1	0
	Lung/ Pleura	3	2	1
Kalikaki et al., 2008	Bone	1	1	0
	CNS	2	1	1
	Lung/ Pleura	1	0	1
Liu et al., 2018	Lung/ Pleura	22	14	8
Luo et al., 2014	CNS	1	0	1
Rau et al., 2016	CNS	8	4	4

Abbreviation: EGFR= epidermal growth factor receptor, CNS= central nervous system
